# Proactive identification of systemic lupus erythematosus: Detailed examination of early signs and symptoms for timely diagnosis

**DOI:** 10.1097/01.NPR.0000000000000276

**Published:** 2025-01-23

**Authors:** Sarah L. Campbell, Nisa K. Patel

**Affiliations:** **Sarah L. Campbell** is an associate clinical professor and FNP in the Sue & Bill Gross School of Nursing at the University of California, Irvine in Irvine, Calif.; **Nisa K. Patel** is an assistant clinical professor and FNP in the Sue & Bill Gross School of Nursing at the University of California, Irvine in Irvine, Calif.

Systemic lupus erythematosus (SLE) is a chronic multisystem disorder characterized by the presence of constitutional symptoms, skin and joint manifestations, hematologic cytopenias, cardiovascular involvement, nephritis, and neurologic indicators that most commonly affects women in their reproductive years between ages 15 and 44 years.^1^ The etiology of SLE is complex and the interaction of environmental triggers and genetic susceptibility is thought to be a fundamental contributor.^1^ The disease process includes the release of type I interferon (IFN-I) and antinuclear antibodies (ANA) that circulate and deposit into tissue and organs throughout the body, activating an inflammatory response and subsequent damage.^2^

The patient in this clinical case was a healthy young female who presented for an annual wellness visit to a university student health center. She had experienced a moderate degree of pain in her hands, wrists, and knees over the previous 3 months intermittently that limited her ability to be physically active. However, she had not sought care for her symptoms; they were raised incidentally during a review of systems as part of her yearly appointment.

Earlier diagnosis and management of SLE have been shown to result in lower incidences of life-threatening disease. Delayed diagnosis must be prevented in younger patients who demonstrate symptoms but may not recognize or seek care for them due to low health literacy, difficulty navigating transitional healthcare, and attenuation of symptoms.^3^ This case underscores the need for NPs to engage in active listening, recognize potential signs and symptoms of autoimmune disease, and consider common conditions in the differential diagnosis, particularly among younger patients who may not recognize the seriousness of their symptoms or seek care.

## PATIENT INFORMATION

This patient was a 20-year-old Hispanic female. The pain in her hands, wrists, and knees was described as constantly “dull and aching” and would flare up for 1 to 2 weeks at a time, indicating a cyclical pattern. She reported that the pain was worse with physical activities such as gripping or grasping items, typing, lifting heavy objects, standing for prolonged periods, and taking long walks across campus. The only treatments she had tried were occasional use of acetaminophen 650 mg and rest, both of which provided minimal relief. Other symptoms mentioned during her visit included dry eyes and slightly elevated stress levels due to academic demands. She reported no other associated symptoms. Her medical history included heavy menses and childhood asthma. The patient had never smoked and denied use of recreational drugs and alcohol. She had never been sexually active and had never been on any regularly prescribed or over-the-counter medications other than the “as needed” acetaminophen. Pertinent family medical history included a paternal grandmother who had SLE.

## CLINICAL FINDINGS, DIAGNOSTIC ASSESSMENT, AND THERAPEUTIC INTERVENTION

A comprehensive physical exam was completed during the visit with unremarkable findings except very slight swelling of the metacarpophalangeal joints of the second and third digits of both hands with tenderness to palpation. There was no warmth or erythema present. Examination of all other joints including wrists and knees was normal. After careful consideration of her history of present illness, family history, demographics, and physical exam findings, the differential diagnosis included rheumatoid arthritis (RA), SLE, or a connective tissue disorder. She reported that her symptoms had been “less intense” during the week prior to her wellness visit, indicating she was not in an acute flare. Given her clinical picture, the most likely diagnosis at the time of her visit was thought to be RA due to the specific joints involved, and the symmetrical and cyclical pattern of her pain.

A referral to rheumatology was placed, and lab tests were ordered that day including a comprehensive metabolic panel, complete blood cell (CBC) count, thyroid-stimulating hormone, urinalysis, ANA test, rheumatoid factor (RF), anti-cyclic citrullinated peptide (anti-CCP) antibody, erythrocyte sedimentation rate (ESR), and C-reactive protein. Abnormal values included an elevated ESR (36 mm/h) and ANA titer of over 1:320 with a speckled pattern. Her CBC revealed a critical platelet count of 43,000/mcL. She was notified immediately, advised of ED precautions for thrombocytopenia, and instructed to get additional labs including a repeat platelet count drawn in a sodium citrate, non-ethylenediaminetetraacetic acid (EDTA) tube, peripheral smear, and prothrombin time (PT)/partial thromboplastin time (PTT). Her repeat platelet count was confirmed to be 41,000/mcL, her peripheral smear showed enlarged platelets but normal red and white blood cell morphology, and her PT/PTT were prolonged at 22.8 and 50.1 seconds, respectively. Upon review of her labs and considering her physical exam findings, the diagnosis of SLE was given further attention. According to the European Alliance of Associations for Rheumatology (EULAR) and the American College of Rheumatology (ACR), classification criteria for SLE include positive ANA (titer of 1:80 or higher), evidence of at least one clinical feature such as joint involvement, and a score of 10 or more, according to the EULAR/ACR classification criteria as listed in Table 1.^4^ In her case, the elevated ANA titer (greater than 1:320), edematous and tender joints (score of 6), and thrombocytopenia (score of 4) totaled a score of 10. It is important to note that the diagnosis of SLE was not made based on meeting EULAR/ACR criteria. However, this reinforced her clinical picture and prompted additional investigation and early intervention by the rheumatologist.

**TABLE 1.** Classification criteria for systemic lupus erythematosus (SLE)^4^

**Entry criterion** Antinuclear antibodies (ANA) at a titer of ≥1:80 on HEp-2 cells or an equivalent positive test (ever)			
↓			
If absent, do not classify as SLE If present, apply additive criteria			
↓			
**Additive criteria** **Do not count a criterion if there is a more likely explanation than SLE**. **Occurrence of a criterion on at least one occasion is sufficient**. **SLE classification requires at least one clinical criterion and ≥10 points**. **Criteria need not occur simultaneously**. **Within each domain, only the highest weighted criterion is counted toward the total score§**.			
**Clinical domains and criteria**	**Weight**	**Immunology domains and criteria**	**Weight**
***Constitutional*** Fever	2	***Antiphospholipid antibodies*** Anti-cardiolipin antibodies OR Anti-ß2GP1 antibodies OR Lupus anticoagulant	2
***Hematologic*** Leukopenia Thrombocytopenia Autoimmune hemolysis	3 4 4	***Complement proteins*** Low C3 OR low C4 Low C3 AND low C4	3 4
***Neuropsychiatric*** Delirium Psychosis Seizure	2 3 5	***SLE-specific antibodies*** Anti-dsDNA antibody∗ OR Anti-Smith antibody	6
***Mucocutaneous*** Non-scarring alopecia Oral ulcers Subacute cutaneous OR discoid lupus Acute cutaneous lupus	2 2 4 6		
***Serosal*** Pleural or pericardial effusion Acute pericarditis	5 6		
***Musculoskeletal*** Joint involvement	6		
***Renal*** Proteinuria >0.5g/24h Renal biopsy Class II or V lupus nephritis Renal biopsy Class III or IV lupus nephritis	4 8 10		
**Total score:**			
↓			
**Classify as Systemic Lupus Erythematosus with a score of 10 or more if entry criterion fulfilled**.			

§ = additional criteria within the same domain will not be counted;

∗ = in an assay with 90% specificity against relevant disease controls. Anti-β2GPI = anti–β2-glycoprotein I; anti-dsDNA = anti–double-stranded DNA.

Reused with permission from Aringer M, Costenbader K, Daikh D, et al. 2019 European League against Rheumatism/American College of Rheumatology classification criteria for systemic lupus erythematosus. *Arthritis Rheumatol*. 2019;71(9):1400-1412. doi:10.1002/art.40930 (published by John Wiley and Sons).

Two days after her initial visit, the patient returned to the student health center to review her lab results. A focused physical exam revealed no signs of bleeding. Her complex case required careful treatment due to bleeding and hypercoagulopathy risks from thrombocytopenia and SLE-related inflammation, leading to an urgent hematology referral. She was advised to see both the rheumatologist and hematologist promptly. She mentioned she had never visited healthcare providers without her parents and was unfamiliar with the referral process, so it was necessary to explain the next steps and set clear expectations. She also expressed concerns about coverage for additional lab tests, which were no longer part of a routine wellness visit. It was important to help her understand her insurance plan and provide reassurance about the billing process and her financial responsibilities.

The patient was seen by the hematologist and rheumatologist within 11 days and additional labs were ordered to rule out other autoimmune diseases including various connective tissue disorders. She was prescribed prednisone 60 mg P.O. daily for immune thrombocytopenia suspicious for antiphospholipid syndrome (APS) by the hematologist, due to elevated APS antibodies. Upon follow-up with the rheumatologist, the patient reported a favorable response to prednisone. A diagnosis of SLE was confirmed and the patient was placed on hydroxychloroquine 300 mg P.O. daily. The rheumatologist referred her to ophthalmology for surveillance during hydroxychloroquine therapy and for management of dry eyes. She was referred back to the student health center for contraception initiation for heavy menses and advised to avoid estrogen due to her risk of hypercoagulopathy due to SLE.

## FOLLOW-UP AND OUTCOMES

The goal for pharmacologic treatment of SLE is to mediate the immune and inflammatory responses to minimize end-organ damage.^1^ The patient continued to follow up with hematology and rheumatology and was successfully tapered off prednisone. She was eventually started on anifrolumab (an INF-I receptor antagonist) I.V. infusions once per month as an adjunct therapy and continued on hydroxychloroquine 300 mg P.O. daily. She tolerated this treatment regimen well, her symptoms continued to improve, and she was able to resume a moderate exercise routine without limitations. Her thrombocytopenia resolved, and her platelet count returned to normal at a level of 220,000/mcL. Subsequently, she was started on aspirin 81 mg P.O. to minimize the risk of venous thromboembolism. She returned to the student health center for insertion of a levonorgestrel intrauterine device (IUD) and reported experiencing lighter periods within 3 months of the procedure.

## DISCUSSION

The EULAR/ACR criteria are helpful for healthcare providers to document key clinical and diagnostic features of SLE; however, there is limited use for diagnostic purposes due to inconsistent, isolated, and intermittent symptoms.^4^ There is also a lack of cohesive diagnostic tests, which can lead to additional challenges. To diagnose SLE, other diagnoses must be ruled out. In addition, demographics should be considered, since the disease is more prevalent in young women of childbearing age and in Hispanic, Black, American Indian/Alaska Native, and Asian populations.^1^,^4^-^6^ The EULAR/ACR criteria were developed to improve sensitivity, specificity, and early detection of SLE and are now widely used for SLE classification.^1^,^4^

### Race and ethnicity

Hispanic individuals develop SLE more often than the general US population, and some literature findings suggest that Hispanic individuals often develop SLE at a younger age when compared with other ethnicities and have worse disease progression.^5^,^6^ Additionally Hispanic individuals with SLE have higher morbidity and mortality when compared with other ethnic groups with SLE.^5^ Mortality is four times higher in Latina females with SLE when compared with the general population of individuals without SLE.^5^ Early detection, impeccable care coordination between primary care providers and rheumatologists, and timely treatment can ensure that patients with SLE receive appropriate treatment and may help address disparities in SLE-associated morbidity and mortality among Hispanic females in the US.

### SLE-RA

Arthritis and arthralgias are often the earliest manifestations of SLE and occur in about 90% of patients with SLE.^1^ Inflammatory arthritis tends to be migratory, polyarticular, and symmetrical. Arthritis associated with SLE can be moderately painful but rarely causes erosion or deformity.^7^ If deforming or erosive arthritis is present, a condition involving an RA and SLE overlap should be considered.^7^ In patients with predominant arthralgias or arthritis, anti-CCP antibodies may help include or exclude a diagnosis of RA.^6^ RF may not always be beneficial to an RA-SLE diagnosis, as 20% to 30% of people with SLE also have a positive RF. Anti-CCP antibodies, however, have a much higher specificity for RA and may be more useful for distinguishing arthritis associated with RA.^7^

### Thrombocytopenia

Ten percent of patients with SLE are found to have platelet counts less than 50,000/mcL, and thrombocytopenia often precedes SLE diagnosis and worsens during acute flare-up.^8^ Some of the more common causes of severe thrombocytopenia in patients with SLE include immune thrombocytopenia and APS.^8^ Reducing bleeding risk is the goal for thrombocytopenia, and not all patients require intervention since relative risk for bleeding is low for most patients.^9^ Glucocorticoids are often used for those who do require intervention.^8^ However, it is important to consider that SLE leads to a hypercoagulable state; therefore, in patients who have thrombocytopenia, a complex interplay exists between bleeding risk and need for aspirin therapy.

## PATIENT PERSPECTIVE

The patient expressed gratitude for having a healthcare provider who listened attentively and quickly recognized the seriousness of her symptoms, prompting further investigation. She acknowledged that while her diagnosis was overwhelming, her rheumatologist highlighted that it was detected very early in the disease process, making her case unique but treatable. SLE can often go undetected for years, leading to multisystem involvement and a poorer response to therapy. Despite the lifelong challenge posed by SLE, she has managed to successfully complete her courses, maintain an active lifestyle, and feel optimistic about her future.

## CONCLUSION

This case study highlights the importance of a thorough health history, an appropriate physical exam, and the early recognition of common presentations of rheumatologic conditions to prevent delayed diagnosis. Creating a supportive environment that fosters a therapeutic relationship builds trust and increases the likelihood that subtle complaints or symptoms will be noticed. Additionally, it is crucial to consider a patient's health literacy, particularly during transitions from pediatric to adult healthcare.^3^ Providers at university-based healthcare centers often overestimate young adults' ability to navigate the healthcare system, assuming they have a basic understanding of referrals, insurance approvals, and billing processes. This misconception can be dangerous. A vital lesson from this case was the necessity of educating the patient about the implications of her symptoms and ensuring timely specialist consultations. Early detection can lead to decreased morbidity and mortality, improving outcomes for high-risk patients.
